# Current heterosexual marriage is associated with significantly decreased levels of anxiety symptoms among Chinese men who have sex with men

**DOI:** 10.1186/s12888-020-02563-7

**Published:** 2020-04-06

**Authors:** Zuosen Yang, Shuang Li, Rui Zhang, Jun Na, Yanxia Li, Huijuan Mu, Liya Yu, Li Liu, Wei Sun, Guowei Pan, Lingjun Yan

**Affiliations:** 1 Institute of Preventive Medicine, China Medical University, Shenyang, 110122 China; 2 Institute of Chronic Diseases, Liaoning Provincial Center for Disease Control and Prevention, Shenyang, China; 3 Research Center for Universal Health, School of Public Health, China Medical University, No. 77 Puhe Road, Shenyang North New Area, Shenyang, 110122 Liaoning China

**Keywords:** Men who have sex with men, Marriage, Anxiety symptoms

## Abstract

**Background:**

The high heterosexual marriage rate could be a ‘double-edge’ sword for the emotional health of Chinese men who have sex with men (MSM). The aim of this study was to determine if current marriage and breakdown of marriage (divorce) have different effects on the mental health of Chinese MSM.

**Methods:**

Eight hundred seven MSM were recruited using respondent-driven sampling from four cities in northeastern China. Gay-related stressful events (GRSE) were measured using the Gay Related Stressful Life Events Scale; social support was measured by the Social Support Rating Scale (SSRS); and depressive and anxiety symptoms were assessed using the Self-Rating Depression Scale (SDS) and Self-Rating Anxiety Scale (SAS), respectively.

**Results:**

Of the study participants, 13.4% were married to women and 4.5% were divorced. The rates of marriage or divorce were 35.3 and 75.8% for participants 30–39 and > 40 years of age, respectively. The current married MSM had the highest SDS (50.0 ± 10.9) and SSRS (35.8 ± 8.6) levels, but the lowest SAS (38.7 ± 12.1) levels. Divorced MSM had the highest SAS (44.4 ± 9.6) and lowest SSRS (30.8 ± 8.1) levels. Age, GESE number, and HIV infection were predictors for SDS and SAS, and current marriage was associated with significantly decreased level of SAS (β = − 0.136, *P* = 0.001) based on multiple linear analysis.

**Conclusions:**

Current marriage is associated with significantly decreased levels of anxiety symptoms among Chinese MSM. More studies are needed to understand the mechanisms underlying the effects of different marriage status on the emotional distress of Chinese MSM.

## Background

Marriage between males is illegal and traditional Chinese values require boys to “have a son to carry on the family name”, especially after the one child-one family policy was implemented in the early 1970s in China. Among Chinese men who have sex with men (MSM), most are only children who face more stress to honor their duty to their parents and to follow the social norms more than their counterparts in other countries [1, 2]. To avoid the stigma and discrimination associated with homosexuality and to cope with the stressors from family and society, many Chinese MSM marry and conceal their same-sex behaviors from their wives and members of their broader social network [1–4]. Between one-fourth and one-third of Chinese MSM marry women [3, 4] and most keep their homosexuality a secret [1]. The marriage rate is significantly higher in Chinese MSM than MSM in Western countries (3–5%) [5, 6]. Such a choice, however, could be a ‘double-edge’ sword. On the one hand, marrying a woman and having a child would mask homosexual behaviors, meet social expectations, avoid being “outed” to the family, buffer the stressors from parents and family, and avoid experiencing guilt, shame, and anxiety concerning homosexual behaviors [7–9]. A few studies have shown that keeping homosexual behaviors a secret could reduce the frequency of gay-related stressful events (GRSEs) among MSM [10, 11], but no studies have reported if such a defensive choice could reduce the levels of depression or anxiety symptoms for married MSM. On the other hand, this strategy will result in numerous new problems with significant byproducts for the mental health of MSM [7–9, 12]. The high level of internal gender role conflict (GRC) stemming from the desire for male partners while living with a wife without personal enthusiasm, fear about being known or disclosing their homosexual orientation and behaviors, and risks of transmitting HIV to their wives and girlfriends [13] all will increase psychological distress and reduce self-esteem. Several studies have reported depression, anxiety, sexual abuse, and other psychological syndemics among MSM in China [11, 14], only a few studies have assessed associations between high marriage rates and emotional health [15, 16]. Therefore, whether or not married and/or divorced MSM have significantly higher depression and/or anxiety symptoms than un-married MSM has not been established. The present study aimed to determine the following: (1) if current marriage is associated with significantly decreased levels of anxiety symptoms; (2) marriage to a woman may worsen the emotional status, particularly depression symptoms; (3) the dissolution of a marriage will influence the emotional health and increase and/or decrease the levels of anxiety and depression symptoms.

## Methods

### Subjects

The locations of the cross-sectional study areas and selection of MSM were described previously [11, 17]. In brief, 807 MSM who lived or worked in four cities of Liaoning Province in northeastern China were recruited using a standardized respondent-driven sampling (RDS) procedure in 2012. Respondents were included if they had oral or anal sexual relations with another man during the previous 12 months. Each subject was interviewed personally by trained interviewers. Socio-demographic characteristics, marriage status, sexual identity, and disclosure of homosexual identity were recorded using a structured questionnaire [17].

### Definition of variables

The following socio-demographic factors were recorded: age, education (< 10 years, 10–12 years, ≥13 years), sexual identity (homosexual, bisexual, heterosexual, unconfirmed), marital status (single, cohabitation with a female partner, married, divorced), disclosure of homosexual identity to parents or wife (yes/no).

### Anxiety and depressive symptoms

Subjective feelings of depression and anxiety during the week prior to the interview were quantified using the Self-Rating Depression Scale (SDS) [18] and the Self-Rating Anxiety Scale (SAS) [19]. The details of SDS and SAS has been described in previous paper of the study [17]. Depression and anxiety was defined as scores of SDS over 53 and SAS over 50 [20, 21].

### Social support

Social support was measured by the Social Support Rating Scale (SSRS) [22], which contains 10 items and measures 3 dimensions of social support: subjective support (4 items), objective support (3 items), and support-seeking behavior (3 items). SSRS scores were added up to generate a score of 0 to 50, with a higher score indicating stronger social support. The SSRS has been used with a wide range of Chinese populations due to its high reliability and validity, and the 2-monthtest-retest reliability of 0.92.

### Gay-related stressful life events

GRSEs were measured using the Gay Related Stressful Life Events Scale [23], a 12-item scale that measures the occurrence of incidents related to sexual orientation over the past 3 months. The detailed of SDS and SAS has been described in previous paper [17].

### Statistical methods

Data analysis was conducted using SPSS (version 17.0). Analysis of variance (ANOVA) and the Kruskal-Wallis test were used to compare mean values of SAS, SDS, SSRS, and GRSEs by demographic characteristics and marriage status. We used stepwise multivariable linear regression analysis retaining variables with a *p* value < 0.10 to assess which variables were independently associated with SDS and SAS scores. Age, education, income, sexual orientation, disclosure of homosexual identity, number of GRSE, SSRS score, and HIV infection were included in the stepwise selection procedure.

## Results

Table 1 shows that a total of 807 MSM were recruited with the following demographics: 71.3% were < 30 years of age; 40.3% were bisexual; 47.2% were gay; 13.4% were married to women; 4.5% have divorced; 22.9% revealed their homosexual identities to their parents and wives; 26.0% experienced GRSEs in the past 3 months; and 3.5% were HIV-positive. The marriage and divorce rates (MD) increased rapidly with age and decreased significantly with decreasing education years, which were 35.3 and 75.8% for those 30–39 and > 40 years of age, respectively, and were significantly higher in those who were HIV-positive (39.3%) than in those who were HIV-negative (17.1%), and significantly lower in those who had disclosed homosexual status (9.2%) than in those who had not disclosed homosexual status (20.4%).

**Table 1:** Distribution of marriage status of 807 MSM sampled

	N	%	Single		Cohabitation		Married with female		Divorced		M + D		p
			N	%	N	%	N	%	N	%	N	%	
**Age, years**													
18–29	575	71.3	492	85.6	61	10.6	20	3.5	2	0.3	22	3.8	< 0.001
30–39	133	16.5	66	49.6	20	15.0	38	28.6	9	6.8	47	35.3	
40–64	99	12.3	13	13.1	11	11.1	50	50.5	25	25.3	75	75.8	
**Education, years**													
< 10	255	31.6	164	64.3	32	12.5	40	15.7	19	7.5	59	23.1	0.055
10–12	322	39.9	235	73.0	34	10.6	43	13.4	10	3.1	53	16.5	
≥ 13	230	28.5	172	74.8	26	11.3	25	10.9	7	3.0	32	13.9	
**Sexual identity**													
Gay	381	47.2	281	73.8	45	11.8	36	9.4	19	5.0	55	14.4	0.187
Heterosexual	13	1.6	10	76.9	1	7.7	2	15.4	0	0.0	2	15.4	
Bisexual	325	40.3	219	67.4	34	10.5	57	17.5	15	4.6	72	22.2	
Unconfirmed	88	10.9	61	69.3	12	13.6	13	14.8	2	2.3	15	17.0	
**Disclosure of homosexual identity**													
No	622	77.1	433	69.6	62	10.0	97	15.6	30	4.8	127	20.4	0.001
Yes	185	22.9	138	74.6	30	16.2	11	5.9	6	3.2	17	9.2	
**GRSE**													
No	597	74.0	418	70.0	63	10.6	86	14.4	30	5.0	116	19.4	0.168
Yes	210	26.0	153	72.9	29	13.8	22	10.5	6	2.9	28	13.3	
**HIV**													
No	779	96.5	555	71.2	91	11.7	100	12.8	33	4.2	133	17.1	0.020
Yes	28	3.5	16	57.1	1	3.6	8	28.6	3	10.7	11	39.3	
**All**	807	100.0	571	70.8	92	11.4	108	13.4	36	4.5	144	17.8	

*MD* Marriage and divorce, *GRSE* Gay related stressful event

Table 2 shows that the SDS means increased significantly with age and decreased significantly with education level. The SSRS score showed an opposite linear trend, unlike the SAS score. When sexual identity was considered, gay had the highest SDS, SAS, and GRSEs means and heterosexual MSM had the lowest levels. When marriage status was considered, the married MSM had the highest SDS and SSRS and lowest SAS, and divorced MSM had the highest SAS and lowest GRSEs and SSRS. The SDS and SAS means were significantly elevated among MSM with HIV infection. The SAS level was borderline increased, but GRSEs levels were significantly decreased, for those who disclosed their sexual identity to others.

**Table 2:** Comparison of average levels of anxiety, depression, number of gay-related stressful events, and social support by demographic factors

Risk Factor	SDS			SAS			GRSEs			SSRS		
	Mean	SD	*p* value	Mean	SD	*p* value	Mean	SD	*p* value	Mean	SD	*p* value
**Age, years**												
18–29	46.2	11.2	< 0.001	40.5	8.4	0.029	0.4	0.7	0.050	34.2	7.0	0.220
30–39	48.3	11.3		39.7	8.2		0.3	0.6		33.8	8.4	
40–64	52.0	11.1		42.8	13.4		0.2	0.5		32.8	8.7	
P linearity	< 0.001			0.105			0.015			0.090		
**Education, years**												
< 10	49.3	11.1	< 0.001	40.9	9.5	0.577	0.5	1.0	0.369	32.2	7.1	< 0.001
10–12	47.7	11.6		40.2	8.9		0.4	0.8		34.3	7.5	
≥ 13	44.4	10.9		41.0	9.3		0.5	0.9		35.6	7.4	
P linearity	< 0.001			0.975			0.679			< 0.001		
**Sexual identity**												
Gay	47.6	11.3	0.603	41.7	8.9	0.014	0.5	1.0	0.053	33.9	7.5	
Heterosexual	45.6	10.0		39.0	8.3		0.4	1.1		35.2	9.9	
Bisexual	47.4	11.5		39.5	9.2		0.3	0.8		34.2	7.5	
Unconfirmed	45.9	11.5		40.5	10.2		0.4	0.7		33.5	6.5	
**Marriage**												
Single	46.3	11.3	0.003	40.6	8.4	0.003	0.4	0.9	0.319	34.0	7.1	< 0.001
Cohabitation	49.2	11.6		42.2	9.4		0.4	1.0		33.0	7.3	
Married with female	50.0	10.9		38.7	12.1		0.5	1.0		35.8	8.6	
Divorced	49.3	12.5		44.4	9.6		0.2	0.6		30.8	8.1	
**HIV positive**												
Yes	53.4	12.3	0.004	45.6	16.1	0.004	0.6	1.3	0.300	34.0	7.4	0.670
No	47.1	11.3		40.5	8.8		0.4	0.9		34.6	9.3	
**Disclosure of homosexual identity**												
Yes	46.8	11.6	0.472	41.8	10.0	0.052	0.7	1.2	< 0.001	34.1	7.7	0.740
No	47.4	11.3		40.3	8.9		0.4	0.8		34.0	7.5	
**All**	47.3	11.4		40.7	9.2		0.4	0.9		34.0	7.5	

*SDS* Self-rating depression score, *SAS* Self-rating anxiety score, *SSRS* Social support rating score, *GRSE* Gay related stressful event

Table 3 shows that age, GRSE number, and HIV infection were predictors for SDS and SAS, SSRS (β = − 0.242, *p* < 0.001) and higher education years (β = − 0.119, *p* < 0.001) were associated with significantly decreased levels of SDS. Sexual identity as gay (β = 0.076, *p* = 0.031) and current marriage (β = − 0.136, *p* = 0.001) were significant predictors for SAS.

**Table 3:** Multivariable linear regression analysis assessing factors independently associated with SDS and SAS

	SDS				SAS			
	Beta	95%CI		*p* value	Beta	95%CI		*p* value
Age	0.145	0.104	0.274	< 0.001	0.117	0.041	0.207	0.003
GRSE number	0.148	1.431	3.701	< 0.001	0.155	1.217	3.135	< 0.001
HIV positive	0.083	1.131	9.156	0.012	0.097	1.481	8.293	0.005
SSRS score	−0.242	−0.468	−0.268	< 0.001				
Education ≥13 year (Ref < 10 year)	−0.119	−4.639	−1.348	< 0.001				
Gay (ref: HU)					0.076	0.128	2.657	0.031
Current marriage with female (ref: single)					−0.136	−5.775	−1.531	0.001

*HU* Sexual identity as heterosexual or unconfirmed

## Discussion

The rates of MD were 35.3 and 75.8%, respectively, for those 30–39 and 40–64 years of age in our study, which is consistent with previous reports that Chinese MSM have higher marriage and lower divorce rates than their counterparts in Western countries [1–7]. The significantly lower rate of disclosing homosexual status among married (10.2%) and divorced (16.7%) than single (24.2%) and co-habiting MSM (32.6%); the significantly lower levels of recently experienced GRSEs among those who conceal their homosexual status (0.36 ± 0.80) than those who have disclosed their homosexual status to family members (0.68 ± 1.18), might support the notion that married MSM tend to keep their homosexual status as a secret. Furthermore, the high marriage and low disclosure rate, as a defense strategy against external and internal stressors, protect Chinese MSM from experiencing conflicts or harm related to sexual minority stress, which is in agreement with previous findings in China [23] and internationally [10].

Similar to previous findings in China, we observed that Chinese MSM had higher SDS and SAS scores, but lower social support compared to their heterosexual counterparts [19]. Age, HIV infection, and number of experienced GRSEs were independent predictors of depression and anxiety symptoms, and these results are consistent with previous studies. The elderly face increasing challenges, such as isolation, more stressors, and increased health problems and disabilities, making them more likely to develop psychological problems [18, 24]. Recently experienced GSREs are direct stressors and lead to increased depression and anxiety symptoms [25, 26]. HIV infection will make them face serious physical and psychological burdens and threats, rendering them more likely to experience mental health problems [27]. We found better social support is a protective factor for SDS, consistent with the findings that better social and economic status enhances an individual’s ability to cope with stressful life events and were less likely to suffer from mental health problems [28].

The currently married MSM had the highest depression symptoms and social support levels, but the lowest anxiety symptoms, and current marriage was associated with significantly decreased levels of SAS (β = − 0.136, *p* = 0.001) based on multiple linear analysis. The beneficial effect of current marriage on SAS, as well as the significantly decreased GRSE and SAS levels in MSM who did not disclose their sexual identity compared with MSM who did disclose their sexual identity, provides additional evidence that entering into marriage and keeping homosexual status a secret helps them avoid or buffer the internal and external stressors from multiple sources [7, 12], which in turn decrease the levels of anxiety. In contrast, reduced worry and anxiety could also serve as main causes contributing to the higher marriage rates in Chinese MSM. The highest anxiety score and the lowest social support levels in divorced MSM further confirmed the effects of losing the protective effects of marriage.

The significantly elevated SDS levels in married (50.0 ± 10.9) and divorced (49.3 ± 12.5) compared with single MSM (46.3 ± 11.3) provided some evidence that continuously struggling with numerous unique challenges related to heterosexual marriage and strong internal gender role conflict (GRC) would waste their emotional energy in maintaining a highly problematic relationship discordant with their erotic desires, resulting in unhappiness and depression [7, 12]. Current marriage or divorce was not a significant predictor for SDS in multiple analyses, which was consistent with the findings of a previous study in China [16]. Anxiety and depression symptoms usually co-exist and overlap and should be considered as a continuum spectrum of affective symptoms, with the hallmark of anxiety being worry, while lack of energy, motivation, and sadness prevail for depression. Although anxiety and depression symptoms are considered to be the two main psychological health problems of MSM, limited information is available on what symptoms or aspects are more relevant to the marriage status of MSM. We think our results are in agreement with the cognitive consistency theory that heterosexual marriage could reduce the amount of worry and the level of anxiety, but make the MSM unhappy and fraught with problems, and the dissolution of marriage make MSM more anxious [12, 29].

Considering the large number (17.8 million), high marriage rate, growing HIV epidemic, and poor mental health status among Chinese MSM [11–14, 30, 31], it is critical to help MSM navigate a balance between their own needs and the culturally normative social duties as heterosexual men. More efforts are needed to reduce the social stigma toward homosexual behaviors and external pressure to marry, targeting specific HIV-prevention and professional consult services, and skills to cope with marriage-related troubles. Early detection of anxiety and depression symptoms, and implementing pharmacologic and non-pharmacologic treatment could improve their mental health status and provide support for MSM to accept public health services. Nonetheless, more work is needed to validate our findings and to explain the mechanisms behind the “double-edged sword” effects of marriage on the mental health of Chinese MSM, and assess the possible influence of the rapid changes in lifestyle and attitude toward homosexual behaviors and/or marriage in China.

There were some limitations within this study that need to be acknowledged. The temporal misalignment between marriage status (current status), depression and anxiety (in the past week), GRSE (in the past 3 months), and social support (not specified), as well as the self-reported symptoms or events, may bias their associations. The cross-sectional design of this study made it impossible to determine directionality or causality between marriage status and emotional distress. The 807 MSM were selected from 4 cities in northeast China. It is not certain that our findings can be generalized to other regions of China. Previous studies noted that RDS may have included individuals who are more marginalized, have fewer resources, and/or have more time to participate in the study. Attentions should be paid to the possible selections bias when generalizing our findings to the target MSM population.

## Conclusion

Age, HIV infection, and number of experienced GRSEs are independent predictors for depression and anxiety symptoms, and current marriage is associated with significantly decreased levels of anxiety symptoms among Chinese MSM. High marriage rates in Chinese MSM may play an important role in shaping the high HIV epidemic among themselves and to their wives, as well as their mental health. New interventions to control the HIV epidemic and improve mental health should accommodate the personal, social, and cultural needs of different marriage subgroups, such as policy development and environment building, to reduce social discrimination toward homosexual behaviors and marriage/co-habitation, increasing the capabilities to buffer or cope with the unique marriage-related stressors and protect themselves and their family members from psychological harm and HIV infection, screening, and early treatment of depression and anxiety. More studies are needed to understand the mechanisms underlying the effects of different marriage status on the emotional distress of Chinese MSM.

## Data Availability

The data that support the findings of this study are available from Liaoning provincial CDC but restrictions apply to the availability of these data, which were used under license for the current study, and so are not publicly available. Data are however available from the authors upon reasonable request and with permission of Liaoning provincial CDC.

## Abbreviations

MSM: Men who have sex with men
GRSE: Gay-related stressful events
SSRS: Social Support Rating Scale
SDS: Self-Rating Depression Scale
SAS: Self-Rating Anxiety Scale
GRC: Gender role conflict
RDS: Respondent-driven sampling
LNCDCP: Liaoning Provincial Center for Disease Control and Prevention
ANOVA: Analysis of variance
MD: Marriage and divorce rates
SC: Single and cohabitation
HIV: Human Immunodeficiency Virus
